# High-Throughput Surface Modification of Ordered Mesoporous Alumina Enables Structural Stabilization and Selective Chemical Control

**DOI:** 10.3390/nano16040253

**Published:** 2026-02-14

**Authors:** Sarah Bindon, Thomas W. Colburn, Reinhold H. Dauskardt

**Affiliations:** Department of Materials Science and Engineering, Stanford University, Stanford, CA 94305, USA; sbindon@stanford.edu (S.B.);

**Keywords:** aluminum oxide, manufacturing, mesoporous, thin films

## Abstract

Porous ceramic oxides have gained significant interest as components in a wide variety of energy storage devices. Their use, however, is limited by long and high-temperature processing methods. We recently demonstrated Porogen-integrated Rapid Oxidation (PiRO) as a new method to manufacture porous aluminum oxide in significantly shorter times and with substantial manufacturing cost savings, but challenges remain with the resultant porous matrices. First, carbonaceous residue remains in the films after the combustion event, which is necessary to minimize for electronic applications. Second, the porous structure is not stable at elevated temperatures (>250 °C), which are often required for nanocomposite applications of the matrices where filling with a second phase is achieved through high-temperature annealing. Here, we address these challenges by using post-processing treatments, including UV/Ozone, high-temperature nitrogen oven anneals, and oxygen plasma. First, we characterize the treatments’ efficacy in carbon removal using FTIR and measure bulk carbon removal with XPS. Second, we characterize the matrices’ thickness collapse and porosity changes after treatments with ellipsometry. Finally, we use nanoindentation to understand changes in stiffness resulting from the various treatments. By understanding the treatments’ roles in removing carbon from the films and stabilizing the matrix structure, we are able to select optimal post-processing treatments for designing a stable platform for further applications of the mesoporous oxide.

## 1. Introduction

Porous ceramic oxide films have gained significant interest as components of energy storage devices with applications spanning fuel cells [1,2], supercapacitors [3,4], and dielectrics [5]. We recently demonstrated Porogen-integrated Rapid Oxidation (PiRO) as a new method to manufacture porous ceramic oxides with ordered pore structures, leveraging evaporation-induced self-assembly (EISA) coupled with solution combustion synthesis (SCS) [6].

PiRO significantly decreases both the time and costs associated with manufacturing porous aluminum oxides compared to traditional sol–gel methods [7,8,9]. PiRO alumina also requires less time compared to other porous alumina, such as anodic aluminum oxide (AAO) [10] and sputtered alumina [11]. PiRO precursor solutions include a surfactant that assembles into ordered micelles during a short crystallization step at room temperature and a short, low-temperature oven anneal. The micelles are then used as fuel for the combustion reaction that is initiated at an elevated temperature (<250 °C), forming an ordered, porous matrix. The pore size, structure, and degree of order of the resultant porous matrix is easily tunable by changing the surfactant used in the solution precursor, lending the process to a variety of applications that require precise control over porosity.

PiRO also achieves significant time savings compared to other porous alumina. To produce anodic aluminum oxide, typically a two-step method is used, where a first step electrochemical anodization of the bulk aluminum occurs, followed by a second anodization to define final ordered pore structure and thickness

Further applications of the porous matrix may require filling with a second phase to design nanocomposites. Devices, such as nanocomposite capacitors, can be manufactured by infiltrating a ceramic matrix with a polymer. The tunable nanocomposites can also be used as platforms for polymer nanoconfinement studies to investigate the role of three-dimensional confinement on molecular mobility and stiffening.

The ability to fill the tunable, porous matrix is critical to open the nanocomposite application space, but filling with a second phase requires elevated temperatures (>250 °C). Despite the advances offered by PiRO for mesoporous film manufacturing, challenges remain with the resultant matrix as the matrix can collapse at elevated temperatures. Additionally, the matrix contains residual carbonaceous species in the pores after the combustion event, which are necessary to minimize, as even small changes in total carbon contamination can significantly alter device performance [12,13].

**Figure 1:**
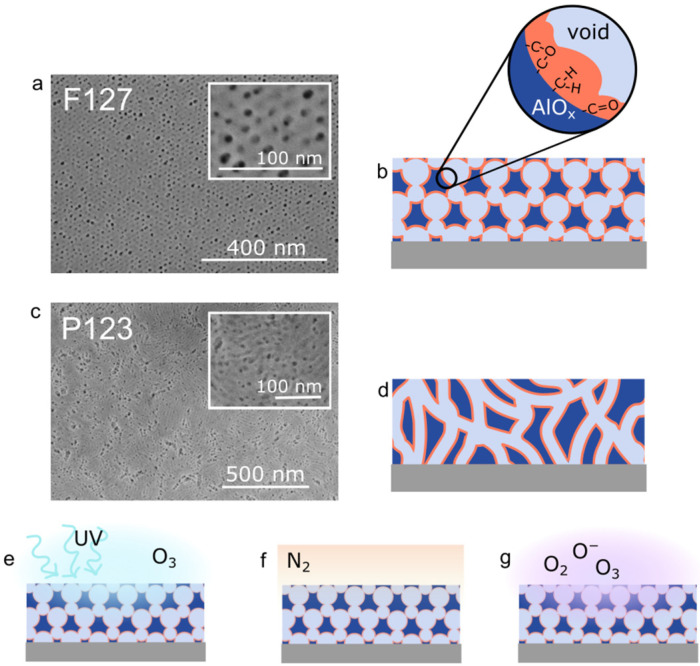
Matrix structures and post-processing treatments. (**a**) SEM image of as-formed PiRO alumina with F127 as surfactant and (**b**) corresponding structure with carbonaceous residue (orange) in pores (light blue) of alumina (dark blue) matrix. Structures presented are representative schematics of results from GISAXS [6]. (**c**) SEM image of PiRO alumina with P123 as surfactant and (**d**) corresponding worm-like chain structure. Post-processing treatments studied for efficacy in removing carbon and stabilizing matrix: (**e**) UV/ozone, (**f**) nitrogen oven anneal, and (**g**) oxygen plasma.

To create a stable platform for nanocomposite design and enable device integration, we use post-processing treatments on the combusted mesoporous oxide, including UV/ozone, nitrogen oven annealing, and oxygen plasma, to stabilize the porous structure and remove residual carbon. Two matrices are presented in this work using different surfactants to change the matrix structure: Pluronic F127 and Pluronic P123. Pluronic F127 generates a porous matrix with 7–10 nm diameter spheroidal pores in a semi-ordered, body centred cubic structure [6] while Pluronic P123 results in a matrix with extended cylindrical, worm-like chain pores of diameters 12–15 nm. Both matrices were treated with each stabilizing treatment, as shown in Figure 1, and characterized to identify the optimal methods for creating a stable and more carbon-free matrix that can be used for further nanocomposite studies or device integration.

## 2. Materials and Methods

Materials. Aluminum nitrate nonahydrate (98%+, ACS grade), Pluronic F127, Pluronic P123, aqueous ammonium hydroxide (30–33%, purists), anhydrous ethanol (absolute, 200 proof), and diglycolic acid were purchased from Sigma–Aldrich (St. Louis, MO, USA). Acetone (HPLC grade) and isopropanol (HPLC grade) were purchased from Fisher Scientific (Waltham, MA, USA).

Solution Preparation. Solution precursors were prepared following methods described elsewhere [6], with the molar ratio as follows: 1:0.004:0.2:0.28:26 for Al^3+^:Pluronic F127:diglycolic acid:ammonia:ethanol. For the matrix with Pluronic P123 as the surfactant, the molar ratio of Al^3+^:Pluronic P123 was 1:0.016 to achieve the desired close-packed and worm-like chain pore morphologies. Before solution deposition via spin coating, single-sided polished silicon wafers were cleaned by scrubbing with 1 wt.% Alconox solution and rinsed in deionized water. Substrates were then rinsed with acetone and isopropanol and dried with a nitrogen gun before treatment under UV/ozone (Jelight 144AX, Irvine, CA, USA) for 10 min.

Deposition and Combustion. For film deposition, 80 µL of precursor was spin-coated at 2000 rpm with a 500 rpm/s acceleration for 24 s onto the UV/ozone-activated silicon substrates. The as-spun films were left in ambient lab conditions for 15 min and subsequently transferred to a preheated convection oven at 60 °C for 15 min. The films were then combusted by being placed on a preheated hot plate at ~240 °C for 15 min.

Post-processing Treatments. For film post-processing, UV/ozone (UVO, Woodbridge, ON, Canada), nitrogen oven annealing, both nitrogen oven annealing and UVO sequentially, or oxygen plasma were used. Samples are outlined in Table 1. For UV/ozone treatments, the films were placed in a UVO-Cleaner for either 5 or 10 min. For nitrogen oven treatments, the films were placed in an oven, which was then purged and filled with nitrogen. The oven was ramped at 5 °C per minute from room temperature to 300 °C and was then held at 300 °C for 1 h or 2 h. Films that underwent both UV/ozone and annealing treatments were removed from the nitrogen oven after 1 h at 300 °C and transferred to the UV/ozone for 5 min. For oxygen plasma treatments, films were placed in an Oxygen Plasma Cleaner (Diener Pico, Ebhausen, Germany) under oxygen plasma for 1 or 2 min.

SEM. Samples were imaged in an FEI Magellan 400XRR scanning electron microscope (SEM, Hillsboro, OR, USA). Samples were deposited on silicon substrates and mounted to aluminum stubs with silver paste.

Images were taken in immersion mode with 2.6 kV accelerating voltage, 1.6 kV stage bias, and 6.25 pA beam current, with drift correction and 64 frame integrations. All imaging occurred without scan interlacing or line integration.

FTIR. Transmission FTIR spectra were collected using the Nicolet is50 (Thermo Fisher) with a KBr detector under a nitrogen purge. IR-transparent, undoped silicon wafers were used as substrates, and the substrate signal was removed with background subtraction. For each spectrum, 100 scans were collected with a resolution of 4 cm^−1^.

XPS. XPS data were collected on a VersaProbe III (ULVAC-PHI, Chigasaki, Japan) with an Al Kα source operating at 50 W with a 200 µm spot size. For depth profiling, films were sputtered with an argon ion gun at 3 kV and 2 µA for a total of 16 min, and high-resolution spectra were taken every 2 min. High-resolution spectra were collected at a pass energy of 55 eV with 4 scans for Al 2p, 4 scans for O 1s, 2 scans for C 1s, and 1 scan for Si 2p. For bulk carbon content, films were sputtered with argon at 3 kV and 2 µA for 5 min, and high-resolution spectra were collected at a pass energy of 55 eV with 10 scans for Al 2p, 10 scans for O 1s, and 20 scans for C 1s. Due to the insulating nature of the aluminum oxide films, both ionization and electron neutralizer guns were used and the spectra were charge corrected using the adventitious carbon signal set at 284.8 eV.

Ellipsometry. A Horiba UVISEL ellipsometer (HORIBA, Kyoto, Japan) was used with a 70° incidence angle and data collected from 400 to 800 nm. To fit the data, the thickness and porosity of the films were allowed to vary while all other parameters were fixed, and the porous fraction was calculated based on the Bruggeman effective medium approximation [14]. For alumina, the new amorphous model was used with parameters of 1.56 for $n_{\infty }$, 9.85 for ω_g_, 2.46 for f_j_, 10.4 for ω_j_, and 0.44 for Γ_j_.

Nanoindentation. Nanoindentation was performed using a Nanomechanics iNano system (KLA Instruments, Milpitas, CA, USA) with a Berkovich diamond tip. Tests were conducted using a constant strain rate of 0.2 s^−1^, target load of 50 mN, target depth of 3000 nm, target frequency of 100 Hz, and applied drift correction. Substrate effects were corrected for by using the Hay and Crawford model [15], with a substrate stiffness directly measured by nanoindentation of 166.88 GPa and an estimated Poisson’s ratio of 0.19 used for the substrate and sample.

## 3. Results and Discussion

**Figure 2:**
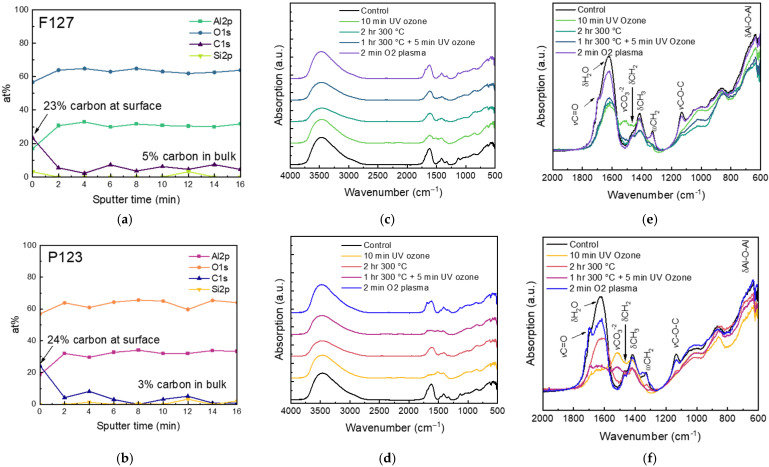
XPS depth profiles of control (**a**), F127 and (**b**), P123 alumina. FTIR spectra of control and post-processed alumina (**c**), F127 and (**d**), P123 alumina, with assigned peaks from 2000 to 600 cm^−1^ for (**e**), F127 and (**f**), P123 alumina.

XPS depth profiling shows that the as-combusted matrices have significant carbon content on the surface (~25 at.%) as well as some residual carbon (~5 at.%) throughout the bulk of the films (Figure 2), making post-processing treatments necessary for reducing carbonaceous contamination in the matrices.

FTIR is used to evaluate the efficacy of various treatments on carbon removal, as shown in Figure 2. The control and treated PiRO films have characteristic peaks of aluminum oxide from 600 to 1000 cm^−1^, including the bending mode of Al−O–Al (δAl–O–Al) from 650 to 700 cm^−1^ and the stretching mode of Al–O (νAl–O) at 850 cm^−1^ [16]. The films also show the broad O–H stretching peak (νO–H) from 3200 to 3600 cm^−1^, as expected for the amorphous alumina and H–O–H bending (δH_2_O) at 1620 cm^−1^ [17]. Though elevated temperature anneals remove physisorbed water, significant hydroxyls persist, which may reuptake water after cooling, resulting in the water peak observed in FTIR conducted at ambient conditions.

FTIR also reveals significant carbon content remaining in the films, which can be removed depending on the post-processing treatment used. The shoulders on the H_2_O bending peak at 1690 cm^−1^ and 1720 cm^−1^ can be assigned to the C=O stretching peak (νC=O), possibly from remaining diglycolic acid in the film [18]. The peak at 1720 cm^−1^ may be due to free carbonyls, while the peak at 1690 cm^−1^ may result from carbonyls bonded to the oxide surface [18]. While oxygen plasma lessens the amount of water in the films, it fails to lessen any of the carbonyl left in the films, unlike the UV/ozone treatment and oven anneal that remove the residual carbonyl. Similarly, UV/ozone and oven annealing prove effective in lessening the peak at 1130 cm^−1^, which is assigned to the C–O–C stretching mode (νC–O–C), possibly also from residual diglycolic acid in the film [19]. The remaining peaks from 1350 to 1500 cm^−1^ can be assigned to –CH_2_ (δCH_2_) and –CH_3_ (δCH_3_) bending modes and are similarly lessened through UV/ozone or oven anneal treatments [18,20].

The UV/ozone treatment introduces a new peak at 1515 cm^−1^, here assigned to –CO_3_^−2^ stretching (νCO_3_^−2^) [21] We postulate that the O_3_ molecules from the UV/ozone treatment oxidize residual organics, forming CO_2,_ which in turn reacts with surface hydroxyls to form carbonate on the aluminum oxide surface [22]. Unlike the UV/ozone, the oxygen plasma operates under a vacuum, so CO_2_ is removed and unable to react with the alumina surface as it does in ambient conditions during the UV/ozone treatment. While most effective at removing residual carbon, the UV/ozone’s formation of carbonate may be negative for porous alumina applications [23], so a shorter treatment time is sufficient.

**Figure 3:**
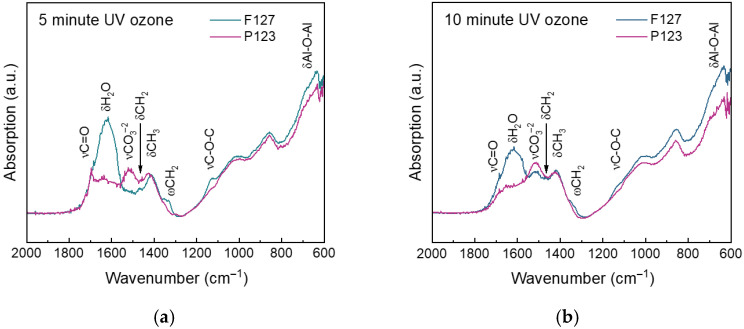
FTIR spectra of matrices treated with UV/ozone for (**a**) 5 min and (**b**) 10 min. For both treatment times, the P123 matrix, with larger pores, is more susceptible to carbonate formation.

Compared to the smaller pore-sized matrix made using the surfactant Pluronic F127, the larger pores of the matrix made using the surfactant Pluronic P123 appear more susceptible to UV/ozone treatment, as shown in Figure 3. The carbonate peak at 1515 cm^−1^ is larger for the same amount of time in the P123 matrix than in the F127 matrix. After oven annealing and a 5 min UV/ozone treatment, the F127 matrix shows negligible carbonate, while carbonate begins to appear in the P123 matrix. Especially for larger pore sizes, restricting the UV/ozone treatment to short times is necessary for minimizing the formation of carbonate.

### 3.1. Structural Characterization of Treated Films

**Figure 4:**
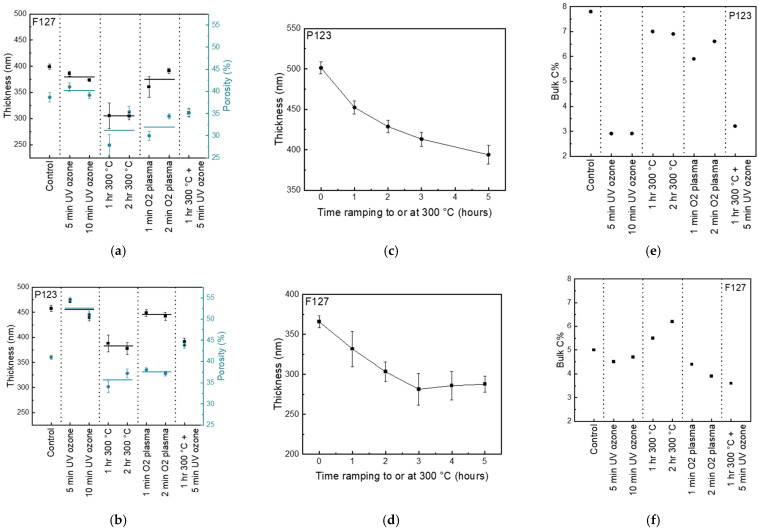
Thickness and porosity of control and post-processed treated alumina modelled from ellipsometry for (**a**) F127 and (**b**) P123 alumina, with average of treatment type shown in solid line. Thickness of (**c**) F127 and (**d**) P123 matrices after increasing time in nitrogen oven modelled from ellipsometry, and bulk carbon content of (**e**) F127 and (**f**) P123 matrices from XPS.

Ellipsometry is used to evaluate the dimension loss of the alumina matrices after treatments. Using a Bruggeman effective medium fitting, the thickness and porosity of the films are modelled, as shown in Figure 4.

Post-processing treatments have similar effects on the thickness collapse of both matrices (Figure 4a,b). Before treatment, the F127 alumina matrix is 400 nm thick with 39% of the matrix being voids. The P123 matrix is thicker at 458 nm and slightly higher in porosity at 41% void. For both matrices, neither the UV/ozone treatments nor the oxygen plasma treatment causes significant thickness loss associated with matrix collapse. The nitrogen oven anneals, however, cause substantial thickness losses of 24% for the F127 matrix and 16% for the P123 matrix, where the thickness loss percentage is defined as the difference between control film thickness and post-processed film thickness divided by control film thickness.

Changes in porosity post-treatments follow similar trends for both matrices, except for the UV/ozone treatment. The oven anneals cause, on average, a 19% decrease in porosity in the F127 matrix from 39% to 32% void. A 13% porosity loss is seen in the larger pore-sized P123 matrix, with oven anneals causing porosity to decrease from 41% to 36%. The decrease in porosity of the oven-annealed matrices follows the loss of thickness in the same matrices, although the matrix seems to densify as the percent void does not decrease proportionally to the thickness loss. Matrix densification may be due to water loss and condensation of Al–OH to Al–O–Al, as seen in the significantly decreased νO–H peak in FTIR in Figure 2c,d for oven annealed films compared to untreated films. Oxygen plasma treatments cause similar collapse processes, though slightly smaller porosity losses for both matrices compared to the oven anneals. The UV/ozone does not significantly alter the pore fraction of the smaller pore-sized F127 matrix, but it significantly increases the pore fraction of the P123 matrix from 41% to 53% void, possibly due to carbon loss of species trapped in the worm-like chains. Similar to the increased effect of UV/ozone on the larger pores seen in FTIR, ellipsometry also shows that UV/ozone has an increased ability to remove residual carbon from the larger pores. The larger, worm-like chains may have more connected and accessible porosity, while the F127 matrix may have more closed and inaccessible porosity that the reactive species are unable to treat. Overall, the treatment remains effective for removing carbon from the pores, especially for the P123 matrix.

The thicknesses of the alumina matrices stabilize over time with increased durations of nitrogen oven anneals, as seen in Figure 4c,d. After a 1 h ramp to 300 °C and 2 h held at the temperature, the thickness of the porous alumina begins to remain level for both matrices. Ensuring the alumina matrix is stable and does not continue to change in thickness is critical for its use as a platform for polymer confinement studies or nanocomposites that require further high-temperature oven anneals for diffusion of a secondary phase into the matrix. Oven anneals are the most effective to stabilize the matrix from further changes, despite causing the largest thickness loss after 1–2 h.

Both matrix stabilization and minimal carbon contamination are critical to enabling the use of the alumina matrix as a platform for further nanocomposite applications. While FTIR is used to identify the nature of carbonaceous residue that is removed using post-processing treatments, XPS is used to measure total carbon content. The bulk carbon content, as a percentage of total atomic concentration, can be seen in Figure 4e,f. As similarly seen in FTIR, the UV/ozone treatments are most effective in reducing carbon content, especially for the larger pore-sized P123 matrix. The oven anneals do not significantly lessen carbon content, and the oxygen plasma treatments’ effect on carbon content is minimal (Figure 4e,f). To combine the matrix stabilization from the oven, anneal with the carbon removal from the UV/ozone treatment; then, a 1 h oven annealing happens at 300 °C before a 5 min UV/ozone treatment is employed. For both matrices, the combined treatment’s effect on thickness and porosity is comparable to that seen after oven annealing, with the larger pore-sized matrix porosity increasing slightly due to the UV/ozone’s effect. By combining treatments, the porous alumina is more resistant to dimension losses and less contaminated with carbon, making it better for further use in filling applications.

### 3.2. Mechanical Characterization of Treated Films

**Figure 5:**
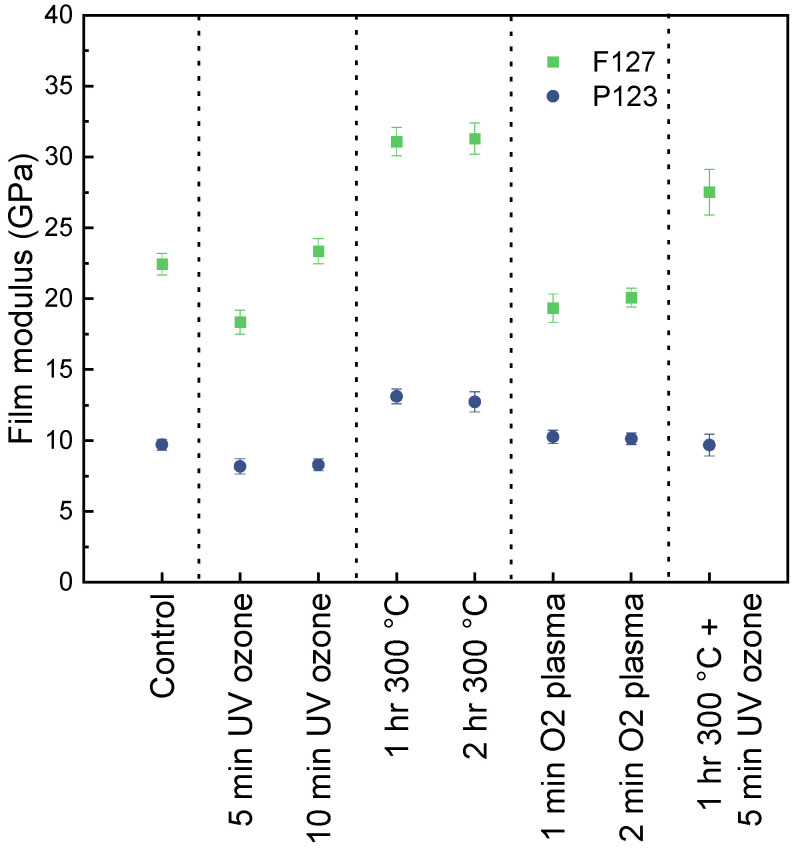
Corrected film moduli of matrices from nanoindentation show higher stiffness of ordered F127 matrix compared to disordered P123 matrix.

To further assess the stability of the porous matrix after treatments, nanoindentation was used to measure changes in film stiffness. Figure 5 shows the results of nanoindentation after correcting for substrate effects using methods designed by Hay and Crawford [15]. The control films have moduli of 22 and 10 GPa for F127 and P123 matrices, which are in line with other reported values for porous aluminum oxide and other ceramics [24,25,26]. Though only slightly higher in porosity, the P123 matrix with disordered, worm-like chains is significantly less stiff than the more ordered F127 matrix. Ordered porosity has been shown to distribute loading more effectively than disordered porosity, thus making the matrix stiffer and more resistant to deformation [24].

No significant changes in film modulus from the UV/O or oxygen plasma treatments are observed. Oven annealing, however, stiffened both matrices as compared to the controls, which further supports that the matrix densifies from annealing, as also seen in significant thickness loss in ellipsometry.

## 4. Conclusions

PiRO is used to manufacture tunable, nanoporous aluminum oxide, but additional treatments of the resultant matrices are necessary to enable a platform for further device integration or nanocomposite studies. Both device operation and diffusion of a second phase into the matrix to create nanocomposites require high temperatures, so it is necessary to stabilize the matrix using a high-temperature oven anneal. Using ellipsometry, we observe that after 1 h at 300 °C in a nitrogen oven anneal, the matrix collapses 15–25% in thickness, but further time at the elevated temperature does not continue to cause significant matrix collapse. Removing carbon from the matrix and pores is also critical for functioning electronic devices. We observe in FTIR that carbon contamination is lessened using a UV/ozone treatment, and XPS is used to further show that UV/ozone minimizes carbon contamination below the detection limit of the technique. Combining the two treatments of an oven anneal and UV/ozone, the carbon content throughout the bulk of the matrix decreases from 5–8 at% to 3–3.5 at% for both matrices. By combining a high temperature oven anneal and UV/ozone treatment, the porous aluminum oxide matrix is best prepared as a platform for further studies as a mesoporous oxide or for nanocomposite device applications.

## Figures and Tables

**Table 1:** Summary of post-processing treatment conditions.

Treatment	Duration
Control	-
UV/ozone	5 min
	10 min
300 °C N_2_ oven anneal	1 h
	2 h
O_2_ plasma	1 min
	2 min
UV/ozone + 300 °C N_2_ oven anneal	5 min UV/O followed by 1 h oven anneal

## Data Availability

The original contributions presented in this study are included in the article. Further inquiries can be directed to the corresponding author.
